# Supplementary material for: Core Bacterial Taxa Determine Formation of Forage Yield in Fertilized Soil
Source: Microorganisms. 2024 Aug 15;12(8):1679. doi: 10.3390/microorganisms12081679 (PMC11356994; doi:10.3390/microorganisms12081679)
Supplement: Supplementary file 1 [file microorganisms-12-01679-s001.zip › microorganisms-3140775-supplementary.pdf]

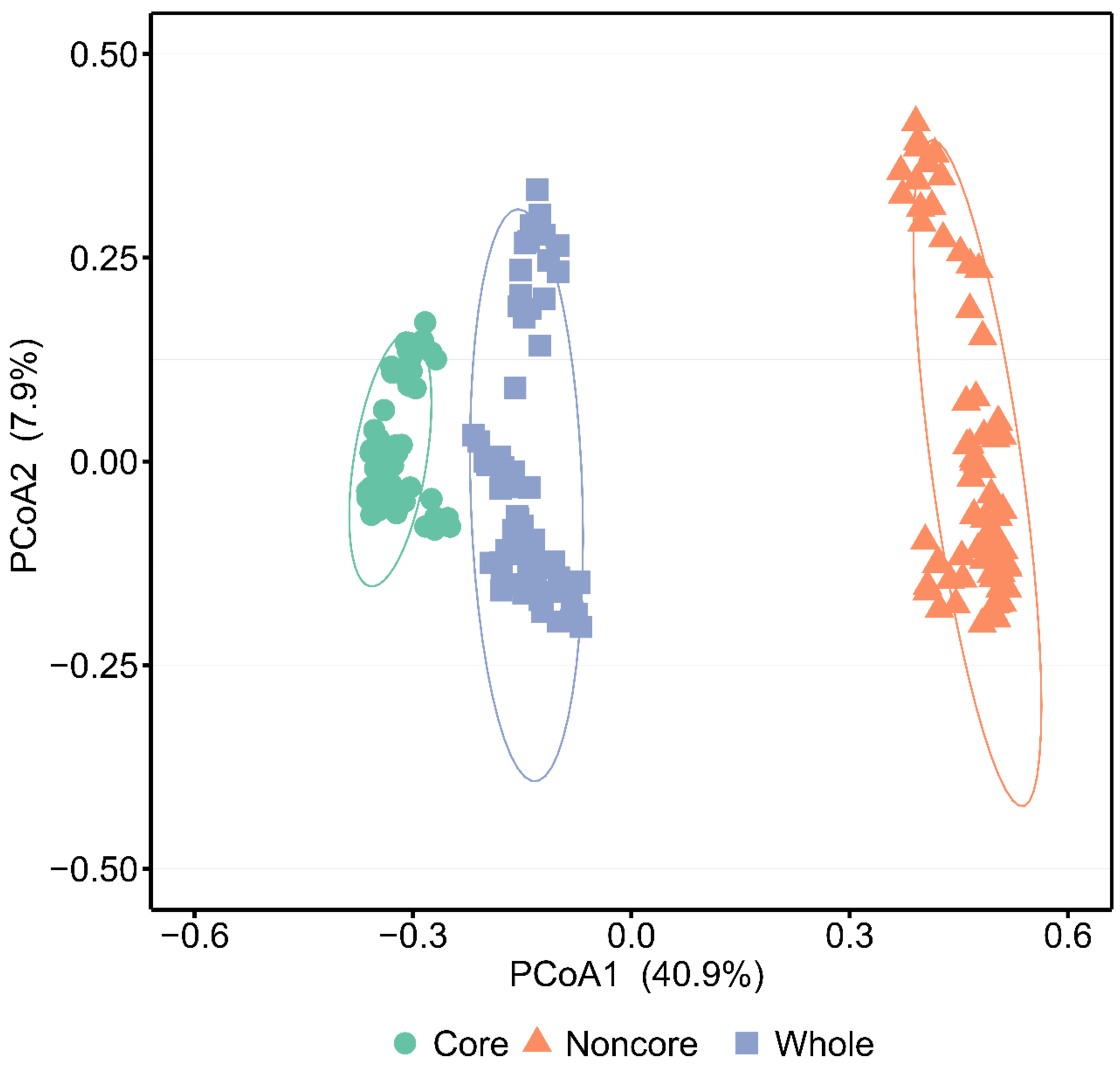

Fig. S1 Principal coordinates analysis for bacterial communities from different fertilization treatments. Whole: all bacterial taxa; Core: Core taxa, and; Noncore: noncore taxa.

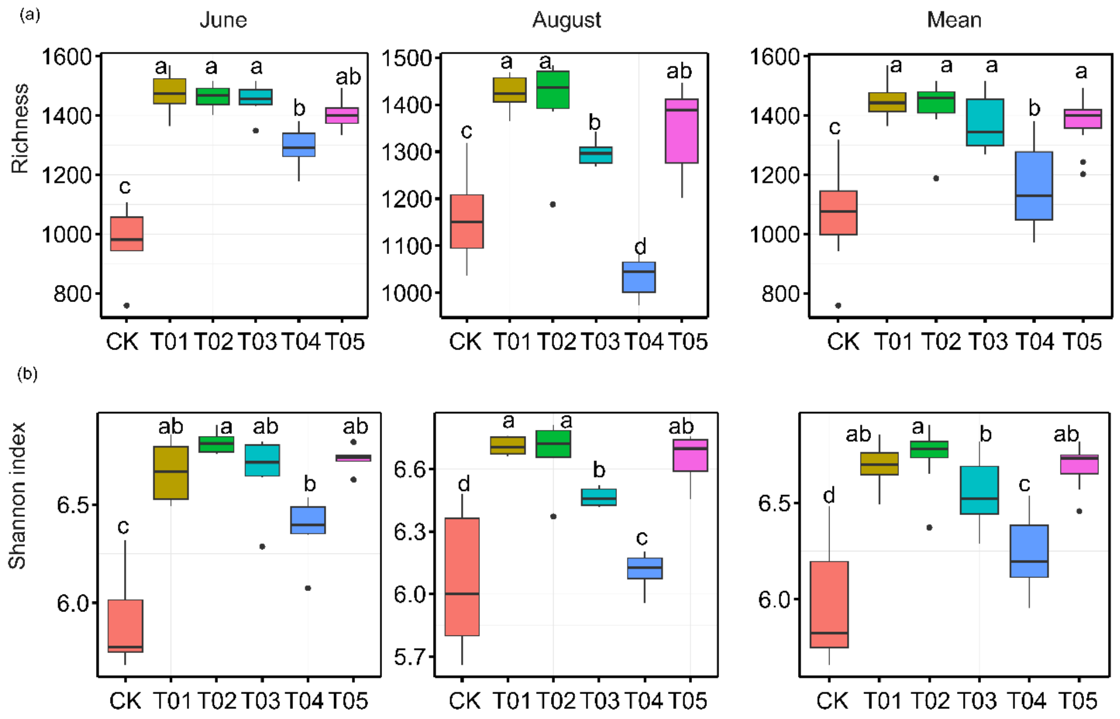

Fig. S2 Alpha diversity of noncore bacterial taxa associated with the different fertilization regimes and sampling times. Lowercase letters result from a post hoc analysis (Duncan's multiple range test, $p < 0.05$) indicate significant differences between different fertilization treatments at the same sampling time. CK: control with no amendment addition; T01, double the standard rate of organic manure; T02, standard rate of organic manure with N input equal to T04; T03, half the standard rate of inorganic fertilizer plus half the standard rate of organic manure; T04, standard rate of inorganic fertilizer reflecting local practice; and T05, double the standard rate of inorganic fertilizer.

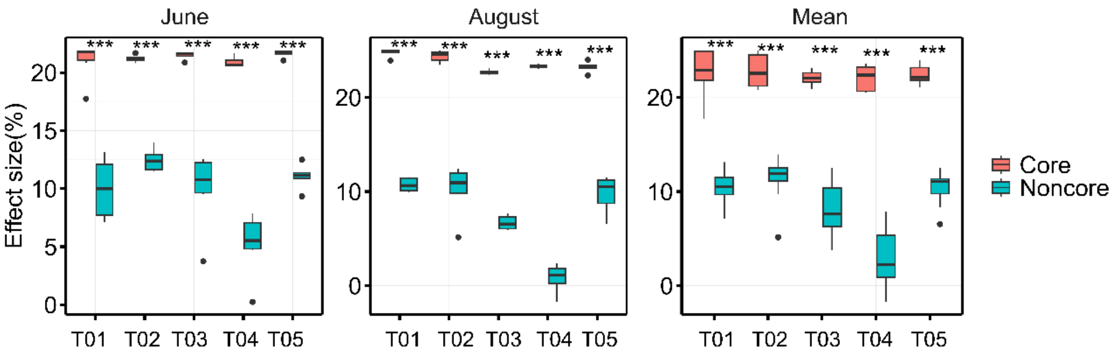

Fig. S3 Showing the effects of fertilization regimes. Values are change in percent (%) and 95% confidence intervals. Effect size= (fertilization regimes - CK)/CK ×100%, ***P < 0.001. CK: control with no amendment addition; T01, double the standard rate of organic manure; T02, standard rate of organic manure with N input equal to T04; T03, half the standard rate of inorganic fertilizer plus half the standard rate of organic manure; T04, standard rate of inorganic fertilizer reflecting local practice; and T05, double the standard rate of inorganic fertilizer.

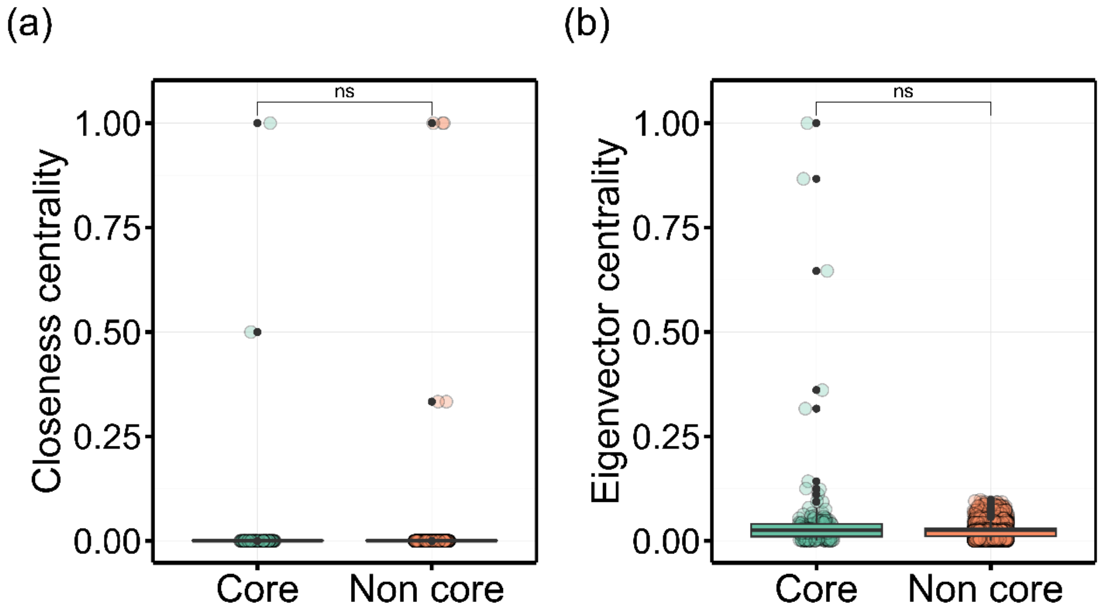

Fig. S4 Unique node-level topological features of core and noncore bacterial taxa in metacommunity co-occurrence networks. ns, not significance.

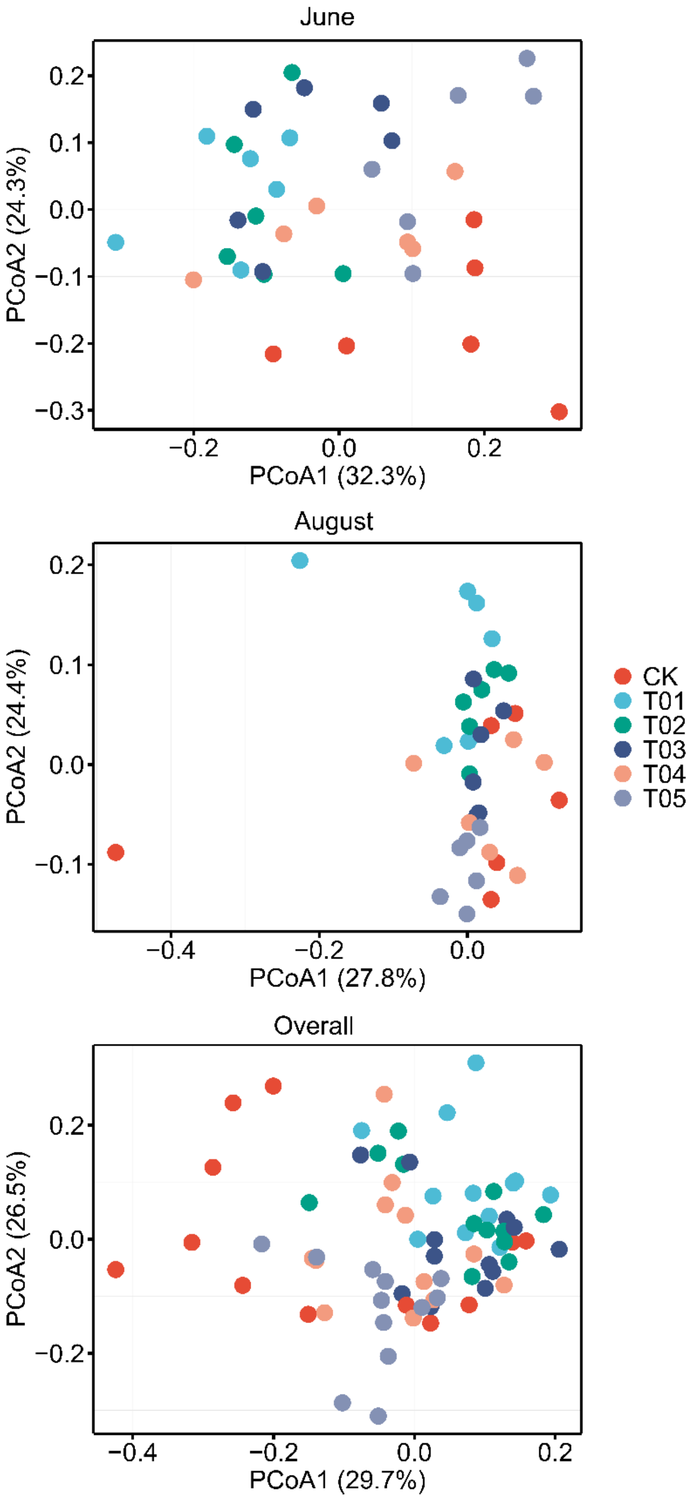

Fig. S5 Principal coordinates analysis showing the clustering of functional characteristics related to carbon and nitrogen cycling of bacterial communities across different fertilization treatments and sampling times. Factors determining the bacterial community variation using PERMANOVA (999 permutations) of Bray-Curtis dissimilarity for the indicated factors. CK: control with no amendment addition; T01, double the standard rate of organic manure; T02, standard rate of organic manure with N input equal to T04; T03, half the standard rate of inorganic fertilizer plus half the standard rate of organic manure; T04, standard rate of inorganic fertilizer reflecting local practice; and T05, double the standard rate of inorganic fertilizer.

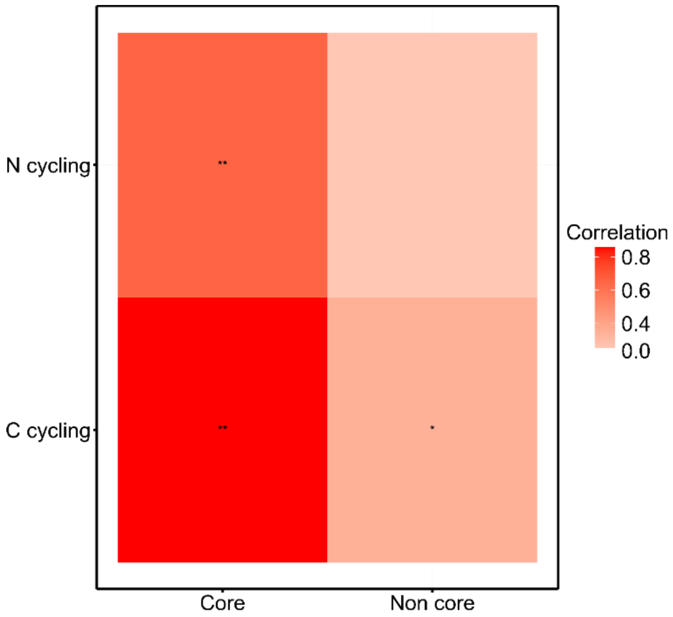

Fig. S6 The relationships between core bacterial taxa and C and N cycling functional characteristics predicated by FAPROTAX database.

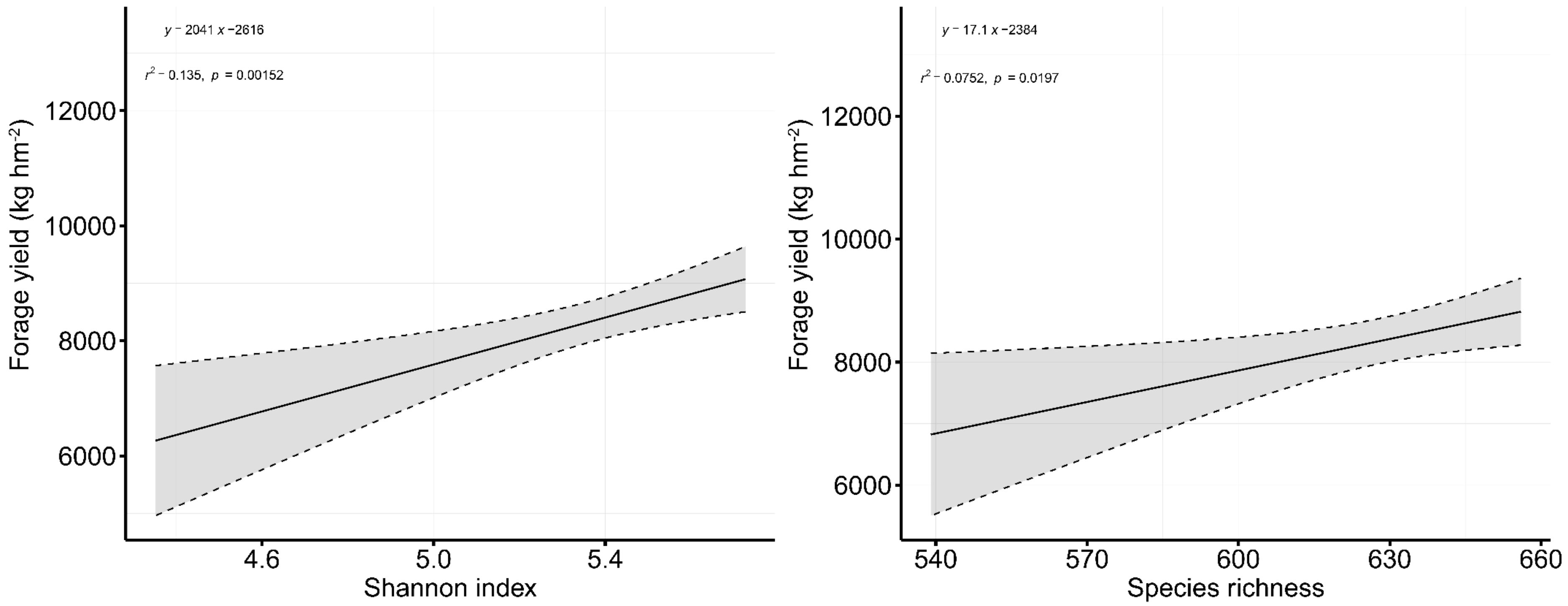

Fig. S7 Ecological relationships between α-diversity of core bacterial taxa and forage yield.
